# Despite the pandemic: upward trajectories of medication adherence and persistence in patients with dyslipidemia

**DOI:** 10.3389/fphar.2024.1488452

**Published:** 2024-12-06

**Authors:** Jieun Jang, Hyun Jung Oh, Eui-Kyung Lee

**Affiliations:** School of Pharmacy, Sungkyunkwan University, Suwon, Gyeonggi-do, Republic of Korea

**Keywords:** COVID-19, pandemic, adherence, persistence, dyslipidemia

## Abstract

**Background:**

Dyslipidemia, a major cardiovascular risk factor, requires consistent medication adherence, but new patients often struggle due to its asymptomatic nature. The COVID-19 pandemic has disrupted global healthcare. This study examined its impact on medication adherence and persistence among Korean patients with dyslipidemia (PWD), comparing the effects on new versus existing PWD.

**Method:**

Nationwide claims data were used to identify PWD and their prescribed medications. Patients were categorized as new or existing PWD and matched 1:1 using propensity scores in both the pre- and post-COVID-19 periods. Medication adherence was measured by the proportion of days covered (PDC), and persistence was assessed by analyzing gaps in continuous medication dispensing. The impact of COVID-19 was evaluated using multiple regression and Cox proportional hazard models.

**Result:**

The pre-COVID-19 cohort included 519,696 patients, and the post-COVID-19 cohort comprised 536,762 patients. PDC significantly increased post-COVID-19, with existing PWD showing a larger increase by 4.74 units (*p* < 0.0001), compared with 2.01 units for new PWD (*p* < 0.0001). Both groups exhibited lower risks of medication discontinuation, with a greater decrease observed in the existing PWD (hazard ratio [HR] 0.780, 95% confidence interval [CI] [0.774–0.786], *p* < 0.0001), compared with the new PWD (HR 0.929, 95% CI [0.923–0.934], *p* < 0.0001). New PWD had fewer annual visits, whereas existing PWD had more visits (both *p* < 0.0001).

**Conclusion:**

Despite the COVID-19 pandemic, medication adherence and persistence improved in both new and existing PWD. Notably, new patients with no prior treatment experience showed weaker positive responses, highlighting the potential need for targeted interventions to support new patients during public health crises.

## 1 Introduction

As a precursor to cardiovascular disease and the leading cause of global mortality, with an estimated 17.9 million deaths in 2019, dyslipidemia poses a significant threat, necessitating proactive interventions and active management strategies (Ha et al., 2015; Kim, 2016; WHO, 2021). Over the past 30 years, the global prevalence of dyslipidemia has substantially increased (Pirillo et al., 2021). This upward trend was also evident in Korea, where the prevalence increased from 8.0% in 2005 to 26.0% in 2021 (KCDA, 2023). Despite the persistent increase in the prevalence of dyslipidemia, only 65% of patients are diagnosed by a physician, with the diagnosis rate falling below 50% among adults aged 30–40 (KCDA, 2023; KSoLA, 2023). This highlights the need for more proactive interventions and management strategies to address dyslipidemia.

Medication adherence is a critical factor in the effective management of dyslipidemia, a chronic disease requiring long-term therapy (Bae et al., 2014; Lee et al., 2015). Notably, the control rates for dyslipidemia treatment can reach 85% when patients consistently take their medications, while patients with low level of adherence are at a significantly higher risk of developing cardiovascular disease than those with high adherence (Jackevicius et al., 2002). Considering the asymptomatic nature of dyslipidemia, which makes it challenging to motivate patients to adhere to treatment, medication adherence in clinical settings is exceedingly poor (Lee et al., 2015; Olmastroni et al., 2020). The proportion of patients with adherence ranges from 22.8% to 79.2%, indicating treatment adherence is sub-optimal (Hope et al., 2019). Particularly for newly diagnosed patients, being new to treatment contributes to lower medication adherence, as they face greater uncertainty about continuing treatment, which often leads to discontinuation (Lopes and Santos, 2021). More than half of those who start treatment for the first time discontinue within 1 year, and only approximately 53.8% receive at least one continuous prescription after initial diagnosis (Brookhart et al., 2007; Maningat et al., 2013). One study reported that among newly diagnosed patients, only 27% of men and 19% of women adhered to their treatment, indicating extremely low adherence (Olmastroni et al., 2020). Additionally, newly diagnosed patients exhibit 32% lower adherence compared to those who have previously been treated (Warren et al., 2015).

Furthermore, the COVID-19 pandemic, a novel infectious disease that emerged in December 2019, has disrupted healthcare utilization among patients worldwide, including Korea (Banerjee et al., 2021; Shin et al., 2021; Olmastroni et al., 2023). In this context, various studies have evaluated the impact of the COVID-19 pandemic on medication adherence among patients with chronic diseases. While these studies have shown that the COVID-19 pandemic significantly impacted medication adherence among patients with chronic diseases, the trend of changes pre- and post-pandemic varied depending on the country and type of disease (Zhang et al., 2020; Mueller et al., 2021; Ismail et al., 2022; Olmastroni et al., 2023). In the case of dyslipidemia, the impact of COVID-19 on adherence has been minimal (Livori et al., 2023; Kardas et al., 2024). However, these studies have generally focused on overall populations without differentiating between new and previously treated patients. This is significant because new patients face greater uncertainty about continuing treatment and could be more susceptible to disruptions, such as the COVID-19 pandemic, making them potentially more affected by external factors that can impact their adherence and persistence.

Therefore, this study aimed to investigate the impact of COVID-19 on disease management in patients with dyslipidemia (PWD) by evaluating medication adherence and persistence using nationwide claims data. Recognizing the heightened uncertainty surrounding treatment adherence in the initial stages of dyslipidemia management, we focused on two distinct patient groups and compared them: 1) those who initiated treatment (new PWD), and 2) those who had a prior history of treatment (existing PWD). By comparing these groups, we can better understand how COVID-19 has affected treatment persistence and identify suitable management approaches for each patient group.

## 2 Methods

### 2.1 Data source

The Health Insurance Review and Assessment Service, a government agency managing South Korea’s National Health Insurance system, maintains a comprehensive claims database of over 50 million individuals. It includes detailed information on the patients’ sociodemographic characteristics, codes for disease diagnosis (International Statistical Classification of Diseases and Related Health Problems, 10th revision [ICD-10]), healthcare utilization (medical facility type), outpatient prescriptions (active ingredients, dosage, and days of medication supplied by the National Health Insurance), and other relevant details. This study used the Health Insurance Review and Assessment Service claims database to select the target population and evaluate the outcomes.

### 2.2 Study design and population

**FIGURE 1:**
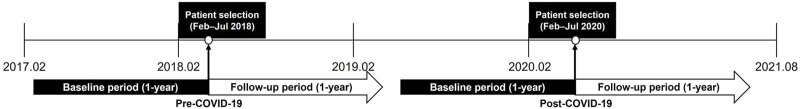
Study design.

This was a retrospective cohort study that utilized the nationwide claims data. The study design is illustrated in Figure 1. Considering that the first confirmed COVID-19 case in Korea occurred on 20 January 2020, the patient selection period for patients post-COVID-19 was from February to July 2020. The corresponding period in 2018 (February–July) served as the patient selection period for patients pre-COVID-19. The index date was defined as the initial prescription date of lipid-lowering agents within the patient selection period. The baseline and follow-up periods for patient inclusion and data analysis were set at 1 year before and after the index date, respectively.

The study population comprised adult patients (aged ≥18 years) who were diagnosed with dyslipidemia (ICD-10 code: E78) as the principal diagnosis during outpatient visits and were prescribed lipid-lowering agents (statins, ezetimibe, fibrates, and omega-3 acids) within the patient selection period. Patients with ischemic heart (ICD-10 code: I20-I25) or cerebrovascular (ICD-10 code: I60-I69) diseases as the principal or secondary diagnoses throughout the study period, including the baseline period, or those who died during follow-up were excluded.

Considering the high treatment uncertainty among newly diagnosed patients (Brookhart et al., 2007; Maningat et al., 2013; Zhao et al., 2020), patients were categorized into “existing” and “new” PWD. Existing PWD were defined as patients with at least one lipid-lowering agent prescription during the baseline period, whereas new PWD were defined as those without any prior lipid-lowering agent prescription within the same period. Consequently, the follow-up periods for all the patient groups (pre-COVID-19 existing PWD, pre-COVID-19 new PWD, post-COVID-19 existing PWD, and post-COVID-19 new PWD) were entirely confined to their respective pre- or post-COVID-19 eras.

To ensure group comparability, existing and new PWD in the pre- and post-COVID-19 periods were matched 1:1 using propensity score-based greedy matching, specifically utilizing the Greedy 8→1 digit matching macro. Propensity scores were calculated using demographic (age, sex, insurance type, and region), clinical (Charlson Comorbidity Index and statin intensity), and healthcare utilization characteristics (medical facility type) as covariates, which have been identified in prior studies as factors influencing medication adherence (Park et al., 2019; Han et al., 2021).

### 2.3 Study outcomes

The outcome of interest was the proportion of days covered (PDC), a commonly used and reliable measure of medication adherence and persistence (Peterson et al., 2007). The PDC was calculated by dividing the total number of days with at least one drug by the total number of days in the follow-up period. It avoids overestimating adherence by counting overlapping medication periods only once, making it suitable for patients switching or taking multiple medications concurrently (Benner et al., 2002). In this study, PDC was calculated as the proportion of days with at least one drug in 365 days. Patients with ≥80% PDC were classified as adherent (Park et al., 2019; Sim et al., 2023). In contrast, persistence measures whether patients continuously took the medication without discontinuation during the follow-up period (Kim and Lee, 2017). Discontinuation, also known as non-persistence, was defined as a gap between refills exceeding half the duration of the prior medication supply. To evaluate the impact of COVID-19 on the changes in patient healthcare utilization patterns, the number of annual outpatient visits and days of medication supplied per visit were analyzed. Analysis of the number of annual outpatient visits and days of medication supplied per visit was limited to claim cases with lipid-lowering agent prescriptions.

### 2.4 Statistical analysis

Categorical variables were analyzed using chi-square (χ^2^) tests to assess their distribution (frequency and percentage). For continuous variables, t-tests were used to compare mean values (standard deviations). Continuous data are presented as means (standard deviations). Kaplan–Meier curves were used to visualize the cumulative persistence rate.

To investigate the impact of COVID-19 and other factors on medication adherence, we employed multiple regression analysis for PDC and logistic regression for the proportion of adherent patients (PDC ≥80% vs*.* PDC <80%). Cox proportional hazards regression was used to assess persistence. Models were adjusted for the following covariates: sex, age, insurance type, medical facility type, region, Charlson Comorbidity Index score, and statin intensity (Park et al., 2019; Han et al., 2021). Considering that the increased number of days of medication supplied per visit observed during the pandemic could potentially affect medication adherence measurement results, we additionally conducted a multiple regression analysis, including it as a covariate (Sim et al., 2023). All analyses were performed using the SAS Enterprise Guide 7.1. All reported *p* values were two-sided, and statistical significance was set at *p* < 0.05. This study was approved by the Institutional Review Board of Sungkyunkwan University (IRB No. SKKU 2023-03-029).

## 3 Results

### 3.1 Study population and patient characteristics

**FIGURE 2:**
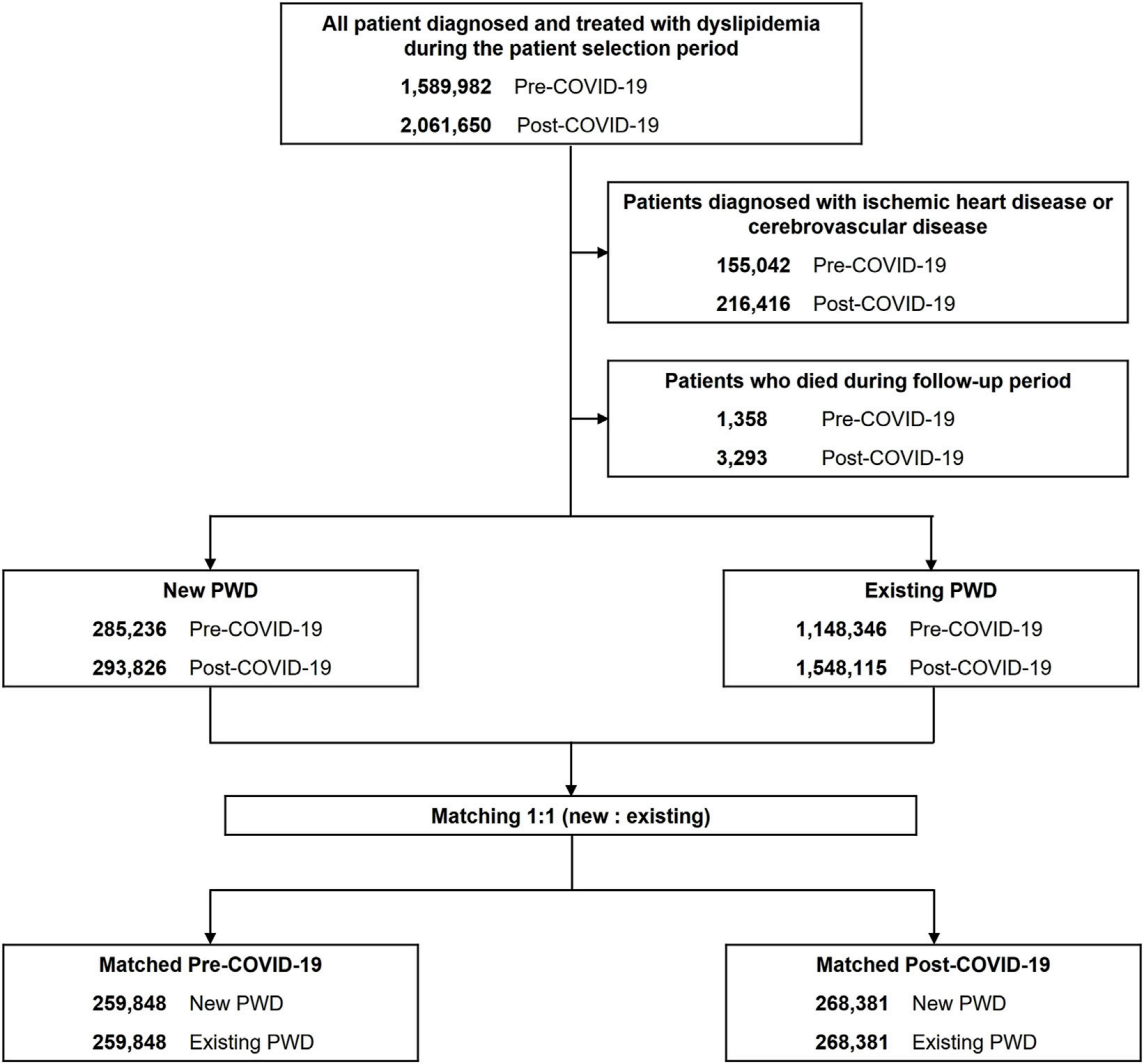
Selection of study participants and construction of propensity score matching PWD, patients with dyslipidemia.

PWD were identified in the pre-COVID-19 and post-COVID-19 periods, respectively (Figure 2). Within these cohorts, 285,236 and 293,826 patients were classified as having new PWD, whereas 1,148,346 and 1,548,115 patients were classified as having existing PWD in the pre-COVID-19 and post-COVID-19 periods, respectively. Following propensity score matching, the pre-COVID-19 cohort included 519,696 and post-COVID-19 cohort comprised 536,762 patients. Table 1 presents the characteristics of new and existing PWD in the pre- and post-COVID-19 periods after propensity score matching. Following propensity score matching, all variables were balanced between the two groups, in addition to statin intensity, in both periods.

**TABLE 1:** Patient characteristics according to patient groups in propensity score matched cohort.

	Pre-COVID-19			Post-COVID-19		
	New PWD (n = 259,848)	Existing PWD (n = 259,848)	p-value	New PWD (n = 268,381)	Existing PWD (n = 268,381)	p-value
Age, mean (SD)	55.7 (11.6)	55.7 (11.5)	0.7806	56.5 (11.8)	56.5 (11.8)	0.9167
<20	65 (0.0)	51 (0.0)	0.0737	13 (0.0)	6 (0.0)	0.4239
20–29	3,620 (1.4)	3,417 (1.3)		4,284 (1.6)	4,125 (1.5)	
30–39	19,755 (7.6)	19,507 (7.5)		18,635 (6.9)	18,708 (7.0)	
40–49	50,088 (19.3)	50,494 (19.4)		46,511 (17.3)	46,496 (17.3)	
50–59	92,781 (35.7)	93,006 (35.8)		89,847 (33.5)	89,948 (33.5)	
60–69	63,317 (24.4)	63,323 (24.4)		73,831 (27.5)	73,935 (27.6)	
70≤	30,222 (11.6)	30,050 (11.6)		35,260 (13.1)	35,163 (13.1)	
Sex, n (%)						
Male	114,778 (44.2)	115,048 (44.3)	0.4508	111,608 (41.6)	111,616 (41.6)	0.9823
Female	145,070 (55.8)	144,800 (55.7)		156,773 (58.4)	156,765 (58.4)	
Insurance type, n (%)						
Health insurance	249,998 (96.2)	250,131 (96.3)	0.3324	258,832 (96.4)	258,923 (96.5)	0.5015
Medical aid	9,850 (3.8)	9,717 (3.7)		9,549 (3.6)	9,458 (3.5)	
Medical facility type, n (%)						
Clinic only	183,449 (70.6)	183,353 (70.6)	0.9223	201,285 (75.0)	201,434 (75.1)	0.8953
Hospital only^a^	39,776 (15.3)	39,772 (15.3)		32,764 (12.2)	32,695 (12.2)	
Mixed	36,623 (14.1)	36,723 (14.1)		34,332 (12.8)	34,252 (12.8)	
Region, n (%)						
Seoul	51,712 (19.9)	51,724 (19.9)	0.7101	57,262 (21.3)	57,419 (21.4)	0.9004
Gyeonggi	74,705 (28.8)	74,881 (28.8)		73,481 (27.4)	73,572 (27.4)	
Metropolitans	60,438 (23.3)	60,091 (23.1)		64,019 (23.9)	63,981 (23.8)	
Others	72,993 (28.1)	73,152 (28.2)		73,619 (27.4)	73,409 (27.4)	
CCI, n (%)						
0	126,087 (48.5)	125,541 (48.3)	0.3026	136,099 (50.7)	135,841 (50.6)	0.7330
1	86,051 (33.1)	86,326 (33.2)		86,920 (32.4)	87,003 (32.4)	
2≤	47,710 (18.4)	47,981 (18.5)		45,362 (16.9)	45,537 (17.0)	
Statin intensity, n (%)						
High	9,454 (3.6)	9,387 (3.6)	0.0043	7,406 (2.8)	7,303 (2.7)	0.1169
Moderate	218,306 (84.0)	217,989 (83.9)		230,446 (85.9)	230,442 (85.9)	
Low	2,410 (0.9)	2,226 (0.9)		2,155 (0.8)	2,023 (0.8)	
No-Statins	29,678 (11.4)	30,246 (11.6)		28,374 (10.6)	28,613 (10.7)	

PWD, patients with dyslipidemia; SD, standard deviation, CCI; charson comorbidity index.

^a^ Patients who have only visited tertiary hospitals, general hospitals, or hospitals.

### 3.2 Impact of COVID-19 on PDC

The PDC exhibited a statistically significant upward trend across pre- and post-COVID-19 periods (*p* < 0.0001) (Table 2). The existing PWD showed a more substantial increase (+5.9%) than the new PWD (+4.0%), resulting in a 1.9% difference in the PDC growth between the two groups. Similarly, the proportion of adherent patients (PDC ≥80%) increased significantly from 31.8% to 38.4% (+6.6%; *p* < 0.0001) in the new PWD and from 56.0% to 66.4% (+10.4%; *p* < 0.0001) in the existing PWD, with a larger increase observed in the existing PWD.

**TABLE 2:** PDC and the proportion of adherent and persistent patients per group.

	Pre-COVID-19	Post-COVID-19	p-value
PDC, mean (SD)			
New PWD	52.8% (33.0)	56.9% (33.8)	<0.001
Existing PWD	73.0% (26.3)	78.8% (23.5)	<0.001
Patients with adherent medication supply (PDC ≥80%), %			
New PWD	31.8%	38.4%	<0.001
Existing PWD	56.0%	66.4%	<0.001
Patients with persistent medication supply, %			
New PWD	24.4%	31.0%	<0.001
Existing PWD	47.0%	57.6%	<0.001

PDC, proportion of days covered; SD, standard deviation; PWD, patients with dyslipidemia.

Logistic regression analysis demonstrated that new PWD in the post-COVID-19 period had 23.4% higher odds of adherence than those in the pre-COVID-19 period (*p* < 0.0001) (Table 3). This increase in adherence was particularly pronounced among existing PWD, with a 48.5% increase in the odds of adherence during the post-COVID-19 period, compared with the pre-COVID-19 period (*p* < 0.0001). Multiple regression analysis (Table 4) revealed a statistically significant increase in the PDC by 2.01 units among new PWD in the post-COVID-19 period, compared with the pre-COVID-19 period. However, the existing PWD exhibited a remarkably larger increase in PDC (4.74 units) during the post-COVID-19 period, compared with the pre-COVID-19 period. While all factors in the new PWD group significantly differed from those in the reference group (*p* < 0.05), only certain factors, excluding sex and statin intensity, showed significant differences in the existing PWD group. Additionally, the β values for covariates affecting PDC were larger in new PWD than in existing PWD, indicating that PDC in new PWD was more sensitive to patient characteristics, whereas PDC among existing PWD remained relatively consistent. Nevertheless, the period (pre-COVID-19 vs*.* post-COVID-19) had a greater impact on changes in PWD.

**TABLE 3:** Logistic regression (adherence determined according to PDC) and Cox-regression (non-persistence determined according to gaps in continuous medications).

	Adherent (PDC ≥80%)			Discontinuation (non-persistent)		
	OR	95% CI	p-value	HR	95% CI	p-value
New PWD						
Pre-COVID-19	Reference			Reference		
Post-COVID-19	1.234	1.219–1.249	<. 0,001	0.929	0.923–0.934	<0.001
Existing PWD						
Pre-COVID-19	Reference			Reference		
Post-COVID-19	1.485	1.468–1.502	<. 0,001	0.780	0.774–0.786	<0.001

PDC, proportion of days discovered; OR, odds ratio; CI, confidence interval; HR, hazard ratio; PWD, patients with dyslipidemia.

**TABLE 4:** Impact of COVID-19 on PDC using multiple regression analysis.

	New PWD		Existing PWD	
	β	p-value	β	p-value
Intercept	5.42	<0.0001	39.37	<0.0001
Age	0.51	<0.0001	0.45	<0.0001
Sex				
Female	Reference		Reference	
Male	−3.12	<0.0001	0.09	0.1938
Insurance type				
Medical aid	Reference		Reference	
Health insurance	−5.02	0.0067	−3.22	<0.0001
Medical facility type				
Clinic only	Reference		Reference	
Hospital only	2.03	<0.0001	2.26	<0.0001
Mixed	−5.15	0.0254	−2.83	<0.0001
Region				
Others	Reference		Reference	
Seoul capital area and Metropolitans	0.54	<0.0001	0.24	0.0009
CCI				
0	Reference		Reference	
1	3.49	<0.0001	1.27	<0.0001
2≤	6.29	<0.0001	2.75	<0.0001
Statin intensity				
Low	Reference		Reference	
Moderate	5.89	<0.0001	1.60	<0.0001
High	6.75	<0.0001	0.38	0.3467
No-Statins	−4.21	<0.0001	−7.13	<0.0001
Days of medication supplied per visit	0.39	<0.0001	0.18	<0.0001
Period				
Pre-COVID-19	Reference		Reference	
Post-COVID-19	2.01	<0.0001	4.74	<0.0001
Adjusted-R^2^	0.1685		0.1263	
p-value	<0.0001		<0.0001	

PDC, proportion of days discovered; PWD, patients with dyslipidemia; CCI; charlson comorbidity index.

### 3.3 Impact of COVID-19 on persistence

**FIGURE 3:**
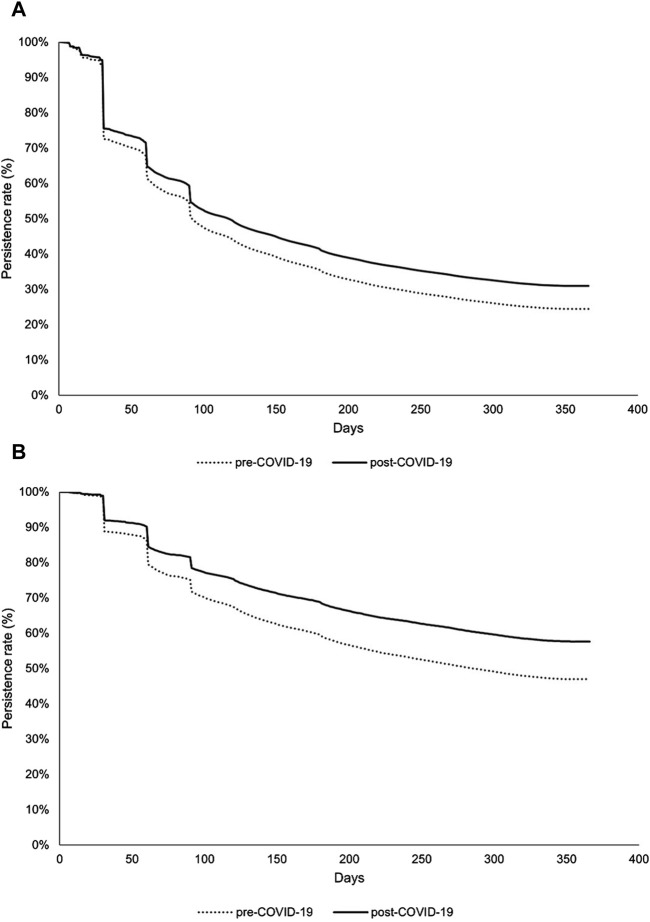
Medication persistence among groups: **(A)** new and **(B)** existing PWD. PWD, patients with dyslipidemia. The log-rank *p*-values for the comparisons between groups in both Figures 3A, B were all less than 0.001.

The proportion of persistent patients demonstrated a statistically significant increase of 6.6% in the new PWD in the post-COVID-19 period, compared with the pre-COVID-19 period (*p* < 0.0001) (Table 2). The PWD group exhibited a substantially larger and statistically significant increase of 10.6% in persistence during the same period (*p* < 0.0001). Consistent with this finding, the Kaplan–Meier survival curve revealed a significant variation in the duration of persistence to medication between pre- and post-COVID-19 in new and existing PWD (*p* < 0.0001) (Figure 3).

Cox regression analysis, with medication discontinuation as the endpoint, revealed a significantly lower risk of discontinuing medication in both new and existing PWD during the post-COVID-19 period, compared with the pre-COVID-19 period (*p* < 0.0001) (Table 3). Specifically, the hazard of non-persistence decreased by 7.1% in the new PWD and 22.0% in the existing PWD.

### 3.4 Impact of COVID-19 on healthcare utilization

The pattern of annual outpatient visits differed between new and existing PWD during the pre- and post-COVID-19 periods. Notably, the new PWD experienced a significant decrease in annual outpatient visits in the post-COVID-19 period, whereas the existing PWD exhibited an unexpected increase in outpatient visits, compared with the pre-COVID-19 period (Table 5). The number of days of medication supplied per visit significantly increased for both new and existing PWD during the post-COVID-19 period, compared with the pre-COVID-19 period. The magnitude of increase was similar between the two groups (new PWD: +3.5 days, existing PWD: +3.8 days).

**TABLE 5:** Healthcare utilization for each group.

	Pre-COVID-19	Post-COVID-19	p-value
No. of annual outpatient visits			
New PWD	5.06 (3.80)	5.03 (3.62)	<0.0001
Existing PWD	6.32 (3.67)	6.42 (3.46)	<0.0001
Days of medication supplied per visit			
New PWD	46.7 (26.2)	50.2 (27.2)	<0.0001
Existing PWD	56.5 (31.3)	60.3 (31.5)	<0.0001

PWD, patients with dyslipidemia Results were presented as mean (SD).

## 4 Discussion

To assess the impact of COVID-19 on dyslipidemia management, this study investigated medication adherence and persistence patterns, differentiating patients with new PWD from those with existing PWD. Analysis of medication adherence, assessed using PDC, and persistence revealed that both new and existing PWD experienced increase in these measures during the post-COVID-19 period, compared with the pre-COVID-19 period, with the increase being more pronounced in the existing PWD. In addition, existing PWD generally showed higher medication adherence and persistence rates, regardless of the COVID-19 pandemic. The number of annual outpatient visits decreased for new PWD increased for existing PWD, compared with the pre-COVID-19 period. The number of days of medication supplied per visit increased for both new and existing PWD, with similar increase observed in both groups.

Regression analysis revealed a significant association between the increased days of medication supplied per visit in both new and existing PWD and improved medication adherence. This finding suggests that changes in physician behavior, such as providing long-term prescriptions during the COVID-19 pandemic, may have been contributing factors (Osawa et al., 2021). This aligns with the result of a previous study that examined the change in the healthcare utilization patterns of patients with hypertension and diabetes in Korea during the COVID-19 pandemic and reported a similar association, suggesting that physician prescribing practices during COVID-19 may have contributed to positive medication adherence (Sim et al., 2023). Additionally, the reports from previous research comparing medication adherence, wastage, and costs between 30- and 90-d refills for chronic conditions, including dyslipidemia, indicate that 90-d refills are associated with improved medication adherence and persistence (Taitel et al., 2012). This supports the notion that more days of medication supplied per visit can enhance adherence in special situations, such as the COVID-19 pandemic. Although long-term prescriptions may improve medication adherence, this approach should be cautiously implemented with standardized protocols to ensure patient safety and treatment efficacy, as it could lead to negative outcomes if patients are left unmanaged for extended periods (Lee et al., 2024).

Both new and existing PWD experienced similar increases in the number of days of medication supplied per visit during the post-COVID-19 period. However, the existing PWD exhibited a greater increase in PDC during the post-COVID-19 period coupled with a lower risk of discontinuation than the new PWD. Moreover, existing PWD were less influenced by demographic, clinical, and healthcare utilization characteristics in terms of PDC than new PWD. This aligns with the findings of studies involving other countries; medication adherence among newly diagnosed PWD in China was poor in the initial 3 months and subsequently declined or remained stable (Zhao et al., 2020). Similarly, Scottish patients initiating statin therapy for secondary cardiovascular disease prevention exhibited a biphasic pattern of adherence, with a high initial discontinuation rate of 12% within 1.5 years, followed by a more gradual decline over the next 6.5 years (Thalmann et al., 2023). Furthermore, a Canadian study highlighted generally low initial persistence rates, with only approximately 53.8% of new patients with dyslipidemia patients receiving at least one continuous prescription after diagnosis (Brookhart et al., 2007). These findings suggest prior treatment experiences among existing PWD may have mitigated the impact of the COVID-19 pandemic on healthcare utilization and medication adherence, compared with new PWD. This aligns with the findings of this study that regardless of the COVID-19 pandemic period, known PWD had higher annual outpatient visits, medication adherence, and persistence than new PWD.

Additionally, annual outpatient visits have increased for existing PWD, in contrast to the decrease observed in new PWD post-COVID-19. Amidst the global decline in healthcare utilization during the COVID-19 pandemic, outpatient visits in 2020 decreased by 15.4%, compared with predictions in Korea (WHO, 2020; Shin et al., 2021). However, this decrease was primarily observed for low-priority illnesses, such as influenza. Notably, healthcare utilization by patients with chronic diseases exceeded the expected value derived from the 2017 to 2019 data in 2020, suggesting minimal pandemic-induced disruption to healthcare access for this population (Oh et al., 2021; Shin et al., 2021). This was because in many countries, lockdown or movement restriction measures were implemented in the early stages of the pandemic, and medical services related to treating chronic diseases were partially or completely suspended. However, in Korea, there was no recommendation from health authorities to postpone or cancel hospital visits during social distancing, allowing patients with chronic diseases to continue using medical services (Lee et al., 2021).

After adjusting for the days of medication supplied per visit, new PWD still demonstrated improved medication adherence and persistence in the post-COVID-19 period, compared with the pre-COVID-19 period. Patients with infectious diseases such as COVID-19 often make healthcare utilization decisions based on a risk-benefit assessment, prioritizing medical care only when the perceived benefits outweigh the potential risks of infection (Lu et al., 2007). This context is particularly relevant for PWD diagnosed during the COVID-19 pandemic, whose initial treatment occurred after the outbreak began. These patients likely focused on managing their condition because of the heightened awareness of health risks.

Our findings suggested that the COVID-19 pandemic had no negative impact on PWD disease management in Korea, which is consistent with the reports of previous studies conducted in various countries. Medication adherence among patients taking statins in Australia was higher during the COVID-19 pandemic than during the pre-pandemic period; however, this difference was not statistically significant (Livori et al., 2023). An Italian study investigating patients taking statins between 2011 and 2020 revealed minimal changes in medication adherence; however, medication persistence significantly decreased (Romagnoli et al., 2022). However, an analysis of statin use in Poland during the COVID-19 pandemic found that prescription and dispensing patterns remained stable even after the COVID-19 outbreak (Kardas et al., 2024). This study revealed a divergent trend: patients with new and existing PWD exhibited improved medication adherence; however, the increase was more pronounced among existing patients. Moreover, existing patients showed increased healthcare utilization after the pandemic. Although the conditions studied were different, this contrasts with the findings of a previous study that analyzed medication prescriptions among German patients with epilepsy during the pandemic. The study found that while medication adherence remained stable for existing patients, it significantly decreased among new patients owing to reduced care (Mueller et al., 2021). In Germany, stringent lockdown measures during the early stages of the pandemic made it difficult for patients to access healthcare services. In contrast, in Korea, flexible government policies ensuring uninterrupted access to the healthcare system and high health consciousness among existing patients may have contributed to these positive outcomes.

This study had several limitations. First, this study classified patients into existing and new PWD based on whether they were previously on medication within 1 year before the index date. Owing to the absence of the duration of illness, the impact of COVID-19 on medication adherence and persistence according to the duration of illness could not be determined. Additionally, medication adherence and persistence were assessed based on the number of prescription days in the claims data, which may not accurately reflect the actual medication intake because patients may not fill prescriptions as prescribed and may not consume all dispensed medications. Finally, this study examined the potential differential impact of the COVID-19 pandemic on new and existing PWDs; however, there were no statistically significant differences between the two groups. Nevertheless, this study provides valuable insights into the potential impact of the COVID-19 pandemic on the management of PWD in South Korea. The observed improvements in medication adherence and persistence among new and existing PWD highlight the importance of public health policies that ensure uninterrupted access to essential healthcare services during crises. Additionally, greater attention should be paid to managing new PWD where uncertainty regarding treatment adherence is relatively high.

## 5 Conclusion

In conclusion, improvements in medication adherence and persistence among both new and existing PWD have occurred despite the unprecedented situation posed by the COVID-19 pandemic in Korea. Notably, PWD who were already undergoing treatment also exhibited increased healthcare utilization post-COVID-19. This is likely attributable to their prior treatment experience, which instilled a greater understanding of the importance of medication and healthcare utilization, thereby mitigating the impact of COVID-19 on their healthcare engagement and medication adherence, compared with the new PWD. Therefore, during public health crises such as the COVID-19 pandemic, new PWD are more likely to be adversely affected, warranting a more focused approach to their management.

## Data Availability

The datasets presented in this article are not readily available because data are available from the first author upon reasonable request and with permission of the Health Insurance Review and Assessment Service. Requests to access the datasets should be directed to Jieun Jang, chocoforjj@gmail.com.
